# Identification of potential suitable areas and conservation priority areas for representative wild animals in the Greater and Lesser Khingan Mountains

**DOI:** 10.1002/ece3.11600

**Published:** 2024-06-18

**Authors:** Chao Zhang, Zhongwei Lu, Hongfei Zhuang, Jiajie Zhou, Yuan Zhang, Xinyu Lv, Minhao Chen, Ali Krzton, Wancai Xia

**Affiliations:** ^1^ National Park (Protected Area) Development Center, National Forestry and Grassland Administration Beijing China; ^2^ Key Laboratory of Southwest China Wildlife Resources Conservation (Ministry of Education) China West Normal University Nanchong China; ^3^ First Institute of Oceanography Ministry of Natural Resources Qingdao China; ^4^ Baimaxueshan National Nature Reserve Diqing China; ^5^ Institute of eco‐Environmental Research Guangxi Academy of Sciences Nanning China; ^6^ Auburn University Libraries Auburn University Auburn USA

**Keywords:** BIOMOD2, conservation priority areas, endangered species, Greater and Lesser Khingan Mountains, potential suitable area

## Abstract

Species geographic distribution and conservation priority areas are important bases for in situ biodiversity conservation and conservation decision‐making. In view of the urgency of endangered species protection, eight representative endangered species in the typical forest ecosystem of the Greater and Lesser Khingan Mountains were studied. Based on 1127 occurrence points and environmental data collected from 2016 to 2021, used BIOMOD2 and Zonation to reconstruct the potential distribution area and identify conservation priority areas of eight species (*Tetrao parvirostris*, *T. tetrix*, *Gulo gulo*, *Alces alces*, *Martes zibellina*, *Moschus moschiferus*, *Lynx lynx*, *Lutra lutra*). The results showed potential distribution areas for almost all species concentrated in the northern part of the Greater Khingan Mountains (GKM) and the central part of the Lesser Khingan Mountains (LKM). The potential distribution areas of each species were as follows: black‐billed capercaillie, 102,623 km^2^; black grouse, 162,678 km^2^; wolverine, 63,410 km^2^; moose, 140,287 km^2^; sable, 112,254 km^2^; Siberian musk deer, 104,787 km^2^; lynx, 139,912 km^2^; and Eurasian otter, 49,386 km^2^. Conservation priority areas (CPAs) clustered in the north GKM and central LKM and totaled 220,801 km^2^, and only 16.94% of the CPAs were currently protected by nature reserves. We suggest that the Chinese government accelerate the integration of existing protected areas in the northern GKM and establish a larger GKM National Park based on cost‐effective multi‐species protection.

## Graphical abstract

Potential suitable areas and conservation priority areas for wild animals in the Greater and Lesser Khingan Mountains.

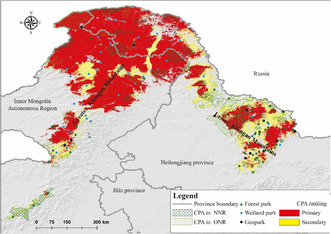

## 1 INTRODUCTION

Since the industrial revolution, habitat destruction, environmental pollution, climate change, and other problems caused by human overexploitation of natural resources have led to an unprecedented decline in global biodiversity (Butchart et al., 2010; Cardinale, 2012; Pimm et al., 2014). The main cause of biodiversity decline has been the destruction of species' habitats (Xia et al., 2020, 2023). Affected by changes in land use, anthropogenic disturbance, and climate change (Barber et al., 2022; Davison et al., 2021), global vegetation biomass has decreased by 50% (Erb et al., 2018), and wild mammal biomass has declined by more than 75% (Bar‐On & Milo, 2018).

Understanding the relationship between species' biogeographical distribution and the environment is the ecological basis for sound policy to protect species and landscapes (Antoine et al., 2013; Brooks et al., 2004; Guisan & Zimmermann, 2000; Pimm et al., 2014). Species distribution models (SDMs) are mathematical tools that predict the distribution probability of target species based on the correlation of species occurrence locations and environmental variables using mathematical algorithms (Elith & Leathwick, 2009; Guisan & Thuiller, 2005; Xia et al., 2023). SDMs are widely used in the fields of endangered species protection (Xia et al., 2020), conservation effectiveness assessment (Tulloch et al., 2016), species reintroduction (Olsson & Rogers, 2009), and species distribution changes over time (Xia et al., 2023; Yang, Chen, et al., 2018; Yang, Zhang, et al., 2018; Zhang, Xia, et al., 2020; Zhang, Yang, et al., 2020).

The identification of conservation priority areas (CPA) for biodiversity is the scientific basis for in situ conservation of species and has become an important planning step for conservation biologists (Brooks et al., 2004; Drummond et al., 2010). Many researchers have analyzed the distribution of large‐scale priority areas for biodiversity conservation, and these studies have played an important role in promoting the construction of the global protected area system (Myers, 1990; Ouyang et al., 2016; Pimm et al., 2014; Yang et al., 2020). Although the theory behind systematic conservation planning is still being developed, there is a consensus in conservation ecology that it is good practice to identify the conservation priority areas of rare and endangered species to carry out in situ conservation of biodiversity. These research targets include single species and multi‐species studies and can involve both plants and animals (Luan et al., 2011; Myers, 1988; Myers et al., 2000).

Species occurrence locations are both a necessary input for SDMs and the basis for systematic conservation planning and policy formulation (Guisan et al., 2013; Hortal et al., 2015). Open access databases have provided effective data support for biodiversity conservation research and were widely used in the fields of conservation biology and ecology (Panter et al., 2020). However, when using these databases, it is difficult to verify the method and time of data collection (Beck et al., 2014). Moreover, due to policy and funding biases, the accuracy of species distribution data is uneven, and species distribution sites are concentrated in areas with low species richness (Beck et al., 2013, 2014; Petersen et al., 2021; Rondinini et al., 2006). So, open access databases often cannot meet the requirements of conservation planning for small‐ and medium‐sized systems (Antoine et al., 2013; Beck et al., 2014). Therefore, it is necessary to clean and expand the data source at the same time (Panter et al., 2020). In the case of unknown species background information, any single data source is insufficient to meet the needs of species conservation planners. Expanding data sources and conducting cross‐verification is an important step for subsequent studies on SDM and the development of conservation policy. At present, many researchers have integrated a variety of data sources to create species profiles, such as local gazetteers, fauna records, infrared camera survey results, field surveys, published monographs and articles, and more for relevant research (Turvey et al., 2015; Xia et al., 2023; Yang et al., 2021; Yang, Chen, et al., 2018; Yang, Zhang, et al., 2018; Zhang et al., 2022; Zhang, Xia, et al., 2020; Zhang, Yang, et al., 2020; Zhao et al., 2018). Due to its powerful algorithm and flexible applications, Zonation has recently become a popular tool in the field of systematic conservation planning. It has proven useful in site selection, boundary optimization, conservation efficiency assessment of protected areas, quantifying the tradeoffs of protection vs. development, and identification of species core habitats (Moilanen, 2007). For example, Zonation was used to integrate the economic costs of conservation into the model when establishing the network scheme of marine protected areas in New Zealand (Geange et al., 2017). It was also employed to construct a systematic conservation scheme for Chinese pangolin (Yang, Chen, et al., 2018; Yang, Zhang, et al., 2018), black grouse (Zhang, Xia, et al., 2020; Zhang, Yang, et al., 2020), Siberian musk deer (Zhang et al., 2022), and canids (Xia et al., 2023) based on historical distributions and conservation cost.

As the largest natural forested area in China and one of the most biodiverse areas in its northern region, the Greater and Lesser Khingan Mountains are key areas for the national nature reserve system. They are also designated as biodiversity conservation priority areas in the China Biodiversity Conservation Strategy and Action Plan (2011–2030). However, in the 1970s, the Greater and Lesser Khingan Mountains used to be the largest logging base in China. In the early 20th century, the logging was stopped and the forest was protected. Due to long‐term deforestation, wetland reclamation, and illegal poaching, a large number of species in the region were once endangered. We selected eight species of rare and endangered terrestrial and semi‐aquatic wildlife (black‐billed capercaillie *Tetrao parvirostris*, black grouse *T. tetrix*, wolverine *Gulo gulo*, moose *Alces alces*, sable *Martes zibellina*, Siberian musk deer *Moschus moschiferus*, lynx *Lynx lynx*, and Eurasian otter *Lutra lutra*) with heavy poaching and abundant distribution data to evaluate the priority areas for biodiversity conservation in the Greater and Lesser Khingan Mountains. In this study, our analysis combines multi‐source occurrence data and SDM to reconstruct their potential distribution areas, then identifies CPAs for these species based on conservation costs, aiming to provide a reference for the planning of natural protected areas in the Greater and Lesser Khingan Mountains.

## 2 METHODS AND METHODS

### 2.1 Study area

**FIGURE 1:**
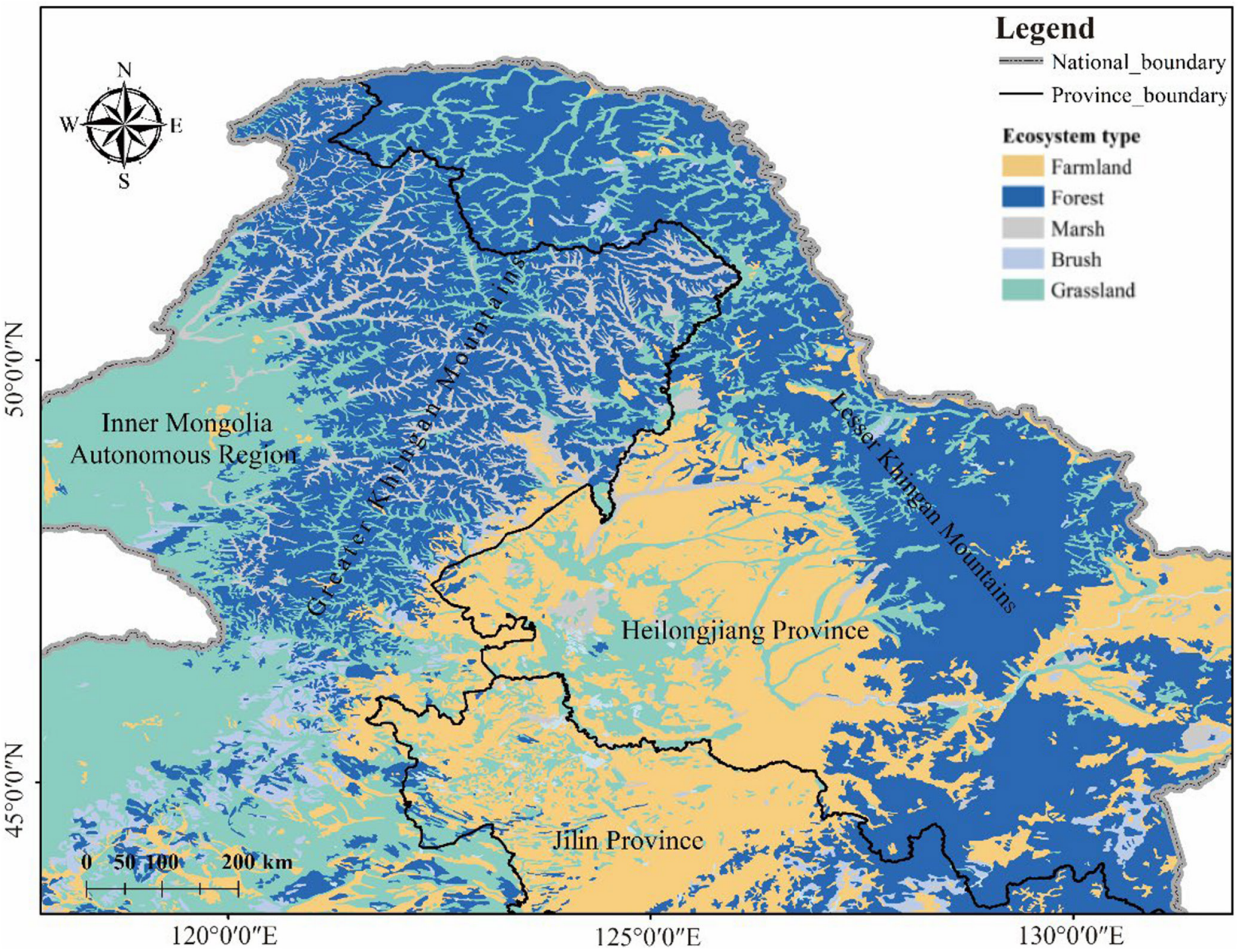
Study area in Northeast China.

The study area is located at 43°22′‐53°17′ N, 117°46′‐131°52′ E in northeast China, with a total area of 1,068,967 km^2^. The Greater and Lesser Khingan Mountains, the largest natural forested area in China, are located in the transition zone between the temperate and cold temperate zones in northern China. The land is dominated by middle and low mountains, hills, and intermountain basins, producing a variety of ecosystems such as forests, grasslands, and wetlands in the area. The main vegetation types are cold temperate coniferous forest, located in the northern part of the Greater Khingan Mountains (GKM), and temperate coniferous broadleaf mixed forest, located in the Greater and Lesser Khingan Mountains (LKM)(Wu, 1980). An important ecological barrier along China's northern frontier, the Greater and Lesser Khingan Mountains play an important role in addressing climate change and protecting biodiversity (Zhao, 1999). While the region's diverse ecosystems and natural conditions have nurtured rich wildlife resources, by the 1990s the wildlife trade and commercial deforestation had led to a sharp decline and retreat of wildlife populations and habitats, leaving some species on the verge of extinction (Yang et al., 2017) (Figure 1).

### 2.2 Species occurrence data investigation

Accurate species distribution data is a prerequisite for species distribution modeling (SDM)(Brooks et al., 2004; Petersen et al., 2021). In order to obtain as comprehensive of a species distribution data set as possible, six surveys (August 2016, August 2019, August 2020, December 2020, January 2021, August 2021) were conducted in the study area between August 2016 and August 2021. Based on published papers, fauna, local chronicles, IUCN species assessment reports, and other data, we excluded areas where the target species were absent, and conducted field surveys of areas where the target species were likely to be distributed (Yang, Chen, et al., 2018; Yang, Zhang, et al., 2018). By means of interviews, questionnaire surveys and field investigations, data collection proceeded in the areas where the target species could possibly occur (Xia et al., 2023; Supplementary Materials D).

In total, we collected 1192 occurrence points for all eight species (the occurrence point number of the eight species see Table 2). The occurrence data obtained from interviews accounted for 40.94% of the points, the questionnaire survey accounted for 12.84%, field surveys accounted for 20.30%, and the literature survey accounted for 25.92%. The number was reduced to 1127 occurrence points after sparse processing using the maximum daily activity radius (see Table 1, Supplementary Materials B).

**TABLE 1:** Types of environmental variables, sources, and species selected.

Type	Environment variables	Data source	Species of variable
Climate	BIO1	Worldclim (http://www.worldclim.org/)	1, 2, 3, 4, 5, 6, 7
	BIO12	Worldclim (http://www.worldclim.org/)	1, 2, 3, 4, 5, 6, 7, 8
	BIO16	Worldclim (http://www.worldclim.org/)	7, 8
	BIO17	Worldclim (http://www.worldclim.org/)	3, 5, 8
	BIO19	Worldclim (http://www.worldclim.org/)	3, 5
	BIO5	Worldclim (http://www.worldclim.org/)	7
	BIO6	Worldclim (http://www.worldclim.org/)	7
	Average precipitation in June	Climate AP v. 2.03	1, 2
	Average temperature in June	Climate AP v. 2.03	1, 2
Vegetation	Birch distribution	Based on vegetation type data simulation http://www.resdc.cn/	1, 2, 3, 4, 6
	Larch distribution	Based on vegetation type data simulation http://www.resdc.cn/	1, 2, 3, 6
	Poplar distribution	Based on vegetation type data simulation http://www.resdc.cn/	2
	Shrub distribution	Based on vegetation type data simulation http://www.resdc.cn/	1, 2, 4, 6
	Forest land density	http://www.resdc.cn/	1, 2, 3, 4, 5, 6, 7, 8
Water	Distance from river	https://www.webmap.cn/	1, 2, 3, 4, 5, 7, 8
	Humidity index	http://www.resdc.cn/	8
Landform	Altitude	SRTM 90 m Digital Elevation Database v4.1	1, 2, 3, 4, 5, 6, 7, 8
	Aspect	SRTM 90 m Digital Elevation Database v4.1	1, 4, 5
	Slope	SRTM 90 m Digital Elevation Database v4.1	1, 2, 5, 6
	Surface relief	http://www.geodoi.ac.cn/	7,8
Anthropogenic disturbance	Distance from the road	https://www.webmap.cn/	1, 2, 3, 4, 5, 6, 7, 8
	Residential density	https://www.webmap.cn/	1, 2, 3, 4, 5, 6, 7, 8
	Human density	http://www.resdc.cn/	1, 2, 3, 4, 5, 6, 7, 8
	Lighting index	http://www.resdc.cn/	1, 2, 3, 4, 5, 6, 7, 8
	Anthropogenic pressure	https://sedac.ciesin.columbia.edu/data/	1, 2, 3, 4, 5, 6, 7, 8

*Note*: BIO1, average annual temperature; BIO5, max. temperature of warmest month; BIO6, min. temperature of coldest month; BIO12, annual precipitation; BIO16, precipitation of wettest quarter; BIO17, precipitation of driest quarter; BIO19, precipitation of coldest quarter; 1, black‐billed capercaillie; 2, black grouse; 3, wolverine; 4, moose; 5, sable; 6, Siberian musk deer; 7, Lynx; 8, Eurasian otter.

### 2.3 Occurrence data vectorization

All species occurrence data were verified against Google remote sensing images to exclude suspicious and unreliable species distribution markers (see Table 2). Spatial autocorrelation is known to reduce the accuracy of SDM. In order to mitigate the effects of spatial autocorrelation on SDM, this study referred to behavioral studies of species to sparsely process the occurrence points of species using the minimum range; that is, one occurrence point within the minimum range is retained at random (Yang et al., 2021). The number of thinned occurrence points for each species is shown in Table 2. With reference to behavioral research and the IUCN species assessment reports, we used the maximum daily activity radius to sparsely process the species occurrence sites. The daily activity radius of each species is as follows: black‐billed capercaillie, 4 km (Gao et al., 1982); black grouse, 1.6 km (Zhang & Li, 1999); wolverine, 10 km; moose, 1.9 km; sable, 2 km; Siberian musk deer, 10 km; lynx, 10 km; and Eurasian otter, 3 km (IUCN, 2021).

**TABLE 2:** Occurrence data, model evaluation, and suitable distribution area of each species.

Species	OP	Data survey sources				DAR (km)	Thinned OP	TSS	AUC	PDA (km^2^)	Modeling selection	Three top environmental factors
		Interview	Questionnaire	Field	Literature							
Black‐billed capercaillie	148	74	15	38	21	4	137	0.955	0.996	102,623	GBM, GLM, RF	Shrub distribution, BIO1, Anthropogenic pressure
Black grouse	141	45	7	13	76	1.6	141	0.954	0.996	162,678	GAM, GBM, RF	BIO1, Anthropogenic pressure, Human density
Wolverine	88	39	5	17	27	10	82	0.985	0.998	63,410	GLM, GBM, RF	BIO1, Residential density, altitude
Moose	145	36	19	45	45	1.9	145	0.955	0.997	140,287	GBM, GLM, RF	BIO1, Anthropogenic pressure, BIO12
Sable	137	39	15	16	67	2	137	0.929	0.993	112,254	GLM, GBM, RF	Anthropogenic pressure, BIO17, Human density
Siberian musk deer	128	61	17	28	22	3	116	0.904	0.988	104,787	MARS, RF, GBM	BIO1, Anthropogenic pressure, Residential density
Lynx	187	52	37	53	45	10	172	0.881	0.987	139,912	GAM, RF, GBM	Anthropogenic pressure, Human density, BIO12
Eurasian otter	218	142	38	32	6	3	197	0.921	0.993	49,386	RF, GAM, GBM	Distance from river, BIO17, humidity index

Abbreviations: DAR, daily activity radius; GAM, generalized additive modeling; GBM, generalized boosting modeling; GLM, generalized linear modeling; MARS, multivariate adaptive regression splines; OP, occurrence points; PDA, potential distribution areas; RF, random forest.

### 2.4 Environment variable database construction

Selecting appropriate environmental variables is crucial for species distribution modeling (Xia et al., 2023). Some researchers believe that selecting more environmental variables with fewer sampling points leads to overfitting, affecting the modeling results (Guisan & Zimmermann, 2000; Williams et al., 2000, 2012). Environmental variables should be screened on the basis of the ecological significance of variables and species (Araújo & Guisan, 2006; Braunisch et al., 2013; Petitpierre et al., 2017; Williams et al., 2012). In addition, when large‐scale species distribution modeling is carried out, temperature and precipitation are important factors affecting species distribution (Jiang et al., 2020). At the same time, June climate is related to breeding success of black grouse (Wegge & Rolstad, 2011). Compared to climate data sets with fewer variables, the 19 bioclimatic variables in WorldClim are widely used without limiting the ability to successfully characterize species distributions (Title & Bemmels, 2018). Considering the ecological habits of the eight representative species (Supplementary Materials A for details), our study compiled a database composed of 25 environmental variables. The database includes nine climate variables, five vegetation‐related variables, two water‐related variables, four variables related to terrain, and five variables related to anthropogenic disturbance (Table 1). The anthropogenic pressure variables are population density, construction land, agricultural land, animal husbandry land, main road, secondary road, light rail, railway, working land, oil wells, wind power facilities, power lines, lights, etc. A total of 13 types of anthropogenic pressure on nature were simulated using the human modification model, which quantified the extent of human impact on the natural landscape on a scale of 0–1 (Kennedy et al., 2019). All variables are continuous variables, and the grid size is defined as 30″ and unified as the WGS‐1984 geographical coordinate system. Since it is difficult to obtain continuous raster data reflecting the distribution of birch (*Betula* spp.), larch (*Larix* spp.), poplar (*Populus tomentosa*), and shrubs, vector data based on vegetation types was used in this study to analyze and calculate vegetation variables by using vegetation simulation methods (Zhang et al., 2022).

### 2.5 Multicollinearity test of environment variables

Collinearity of variables affects the accuracy of modeling. Before modeling, correlation tests of variables should be performed to remove variables with strong correlation and reduce overfitting (Dormann et al., 2013). Compared to the Pearson and Spearman correlation coefficients, which only calculate the correlation between two variables, the variance inflation factor (VIF) combines the explanatory power of all other factors into a single factor, such that the final result only retains the environmental variables with independent predictive ability and does not involve the secondary screening of factors. This is the preferred method to reduce the multicollinearity of environmental variables (Naimi & Araújo, 2016). In this study, the “BiodiversityR” (ver.2.8‐4) package developed in R was used to calculate the VIF between each species and the selected environmental variable (Naimi & Araújo, 2016; Zhang, Xia, et al., 2020; Zhang, Yang, et al., 2020).

### 2.6 Species distribution modeling

Compared with a single model, the combined model provides higher accuracy and reliability (Coetzee et al., 2009; Singer et al., 2018; Thuiller et al., 2009). In this study, the potential distribution of species was estimated using the BIOMOD2 package (version 3.3‐7) in R software (Thuiller et al., 2009; Zhang, Xia, et al., 2020; Zhang, Yang, et al., 2020). The BIOMOD2 package embedded with multiple model algorithms requires both species occurrence and nonoccurrence data. In this study, we refer to the method of generating species nonoccurrence data (Yang et al., 2021), setting 30 km of road as the deviation correction area of background points, and uniformly generating 2000 random points as nonoccurrence data. In order to improve model prediction accuracy, we first used true skill statistics (TSS) and area under curve (AUC) to evaluate the 10 algorithms of BIOMOD2 before combined modeling, selecting the three algorithms with the best performance (see Table 2) to predict the potential distribution of species (Yang et al., 2021). In both model evaluation and combined modeling, we randomly selected 80% of the occurrence data to construct the models and used the other 20% to assess model performance. Thirty repeated iterative operations were carried out for the final combination modeling of each species to reduce the uncertainty of modeling results. The simulation results used the maximum TSS as the threshold to divide the habitat suitability map into distributed/undistributed (Thuiller et al., 2009; Yang et al., 2021). In order to reduce the overestimation of the potential distribution area of species in the model, we removed identified unsuitable areas in the projections (refer to the IUCN habitat description and 2018 land cover data at http://www.resdc.cn/) and used the species minimum range to exclude isolated patches in which the species could not survive.

### 2.7 Identification of conservation priority area based on protection cost

Zonation (version 4.00) was used to identify conservation priority areas based on conservation costs. Zonation produces a highly connected ranking of protection priority areas by removing grids with the lowest protection value one by one (Moilanen, 2007), and outputs 0–1 continuous raster data based on the removal order of the raster. In this study, the core‐area Zonation algorithm was used to prioritize retaining grids with higher suitability to minimize biological loss, while “edge removal rule” was checked to ensure landscape connectivity and reduce edge effects. The warp factor was set to “1”, meaning one grid at a time was removed to ensure optimal operational results. When calculating multi‐species conservation priority areas, we set the species conservation weights according to the conservation levels of the eight representative species: animals with first‐level national protection (black‐billed capercaillie, black grouse, wolverine, moose, sable, and Siberian musk deer) received weight = 2, and those with second‐level protection (lynx and Eurasian otter) received weight = 1. This study uses the nonmonetary measurement method to obtain the cost layer, which is based on data for three normalized environmental variables related to human economic development (human pressure, population density, and settlement density). The Raster Calculator tool in ArcGIS 10.2.2 was used to obtain the cost layer for system protection planning. With reference to the Aichi Biodiversity Target and the requirements of the Post‐2020 Global Biodiversity Framework for biodiversity conservation, the 17% of the total area with the highest conservation value for each species was selected as primary priority, and the area with the next highest conservation value up to 30% was selected as secondary priority.

## 3 RESULTS

### 3.1 Environmental variables and model evaluation

The VIF among the environmental variables for wolverine, moose, sable, and Siberian musk deer was less than 10, so all environmental variables were retained for the SDM. After two iterations, the environmental variable “average temperature in June” was removed for black‐billed capercaillie and black grouse, leaving 16 remaining. For lynx, the environmental variable “annual average temperature” was removed, leaving 13 for SDM. For Eurasian otter, “annual precipitation” was removed, leaving 12 environmental variables for SDM (Supplementary Material C). The most important environmental variables in the SDM by species are shown in Table 2.

The average TSS value of the species combination modeling was higher than 0.881, and the average AUC value was higher than 0.987, indicating good simulation accuracy for the eight representative species (Allouche et al., 2006). The average TSS of every species except for lynx was above 0.9, and the accuracy of the potential distribution prediction for wolverine was highest (TSS = 0.985, AUC = 0.998, Table 2).

### 3.2 Potential distribution area and conservation status of species

**FIGURE 2:**
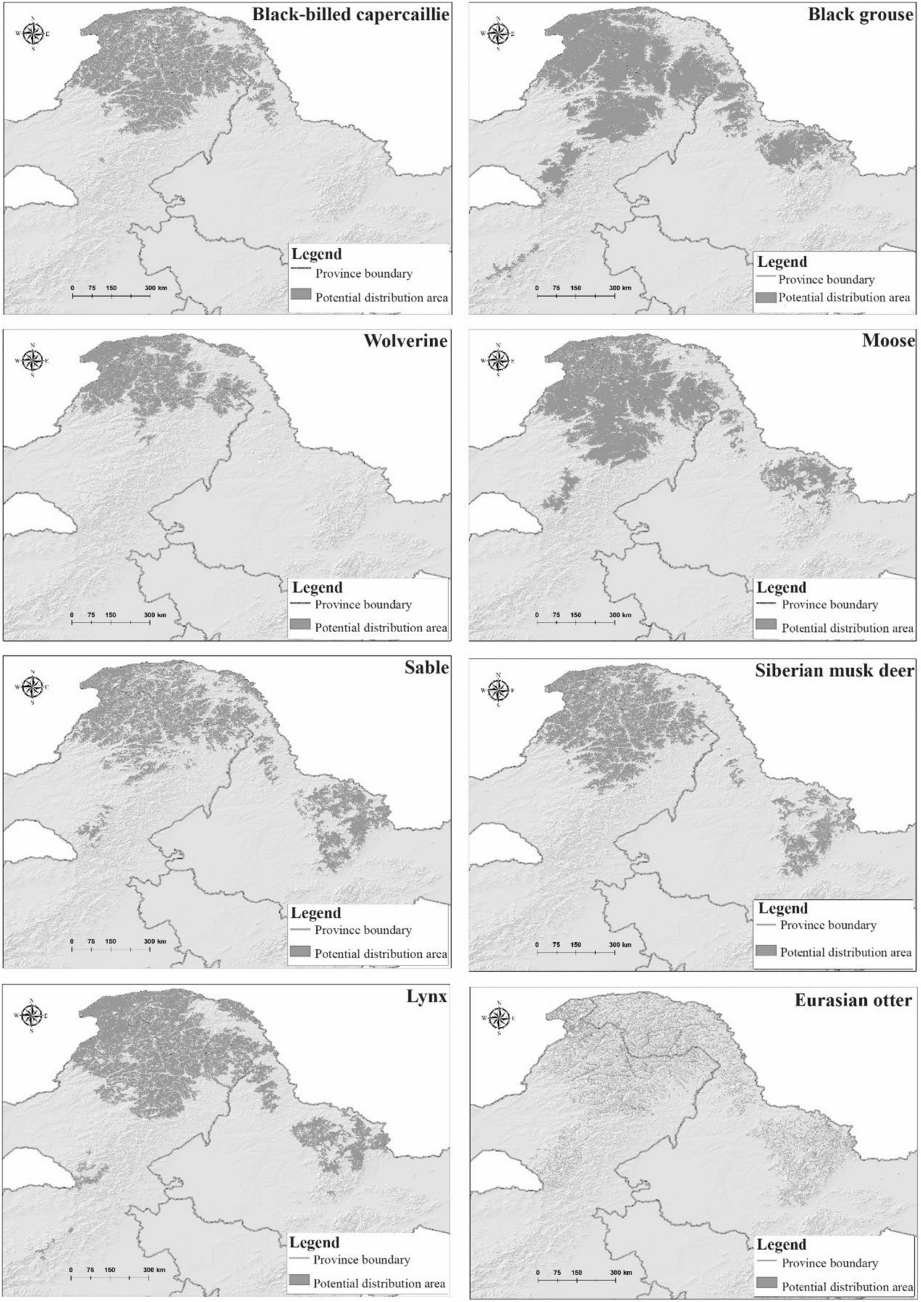
Potential distribution areas of eight representative species in the Greater and Lesser Khingan Mountains.

Black‐billed capercaillie is mainly distributed in the north of the GKM and the northwest of the LKM, with a 102,623 km^2^ potential distribution area (PDA). Black grouse is concentrated in the north of the GKM and the north and central LKM, with a PDA of 162,678 km^2^. The PDA for the wolverine is quite narrow, occurring in the coniferous forests of the cold temperate zone of the GKM north of 50° N latitude with an area of about 63,410 km^2^. Moose were mainly distributed in the north of the GKM and the central LKM, with a PDA of 140,287 km^2^. Sable, widely distributed, occurs mainly in the north of the GKM and in a belt through the middle of the LKM with a PDA of 112,254 km^2^. Siberian musk deer is mainly distributed in the north of the GKM and the southeast of the LKM, with a PDA of 104,787 km^2^. Lynx is distributed in the north of the GKM and the northwest of the LKM, with a PDA of 139,912 km^2^. Finally, Eurasian otter is mainly concentrated in the northern forest of the GKM and the central LKM, the PDA is 49,386 km^2^ (Table 2; Figure 2).

### 3.3 Multispecies conservation priority areas and nature reserve coverage

**FIGURE 3:**
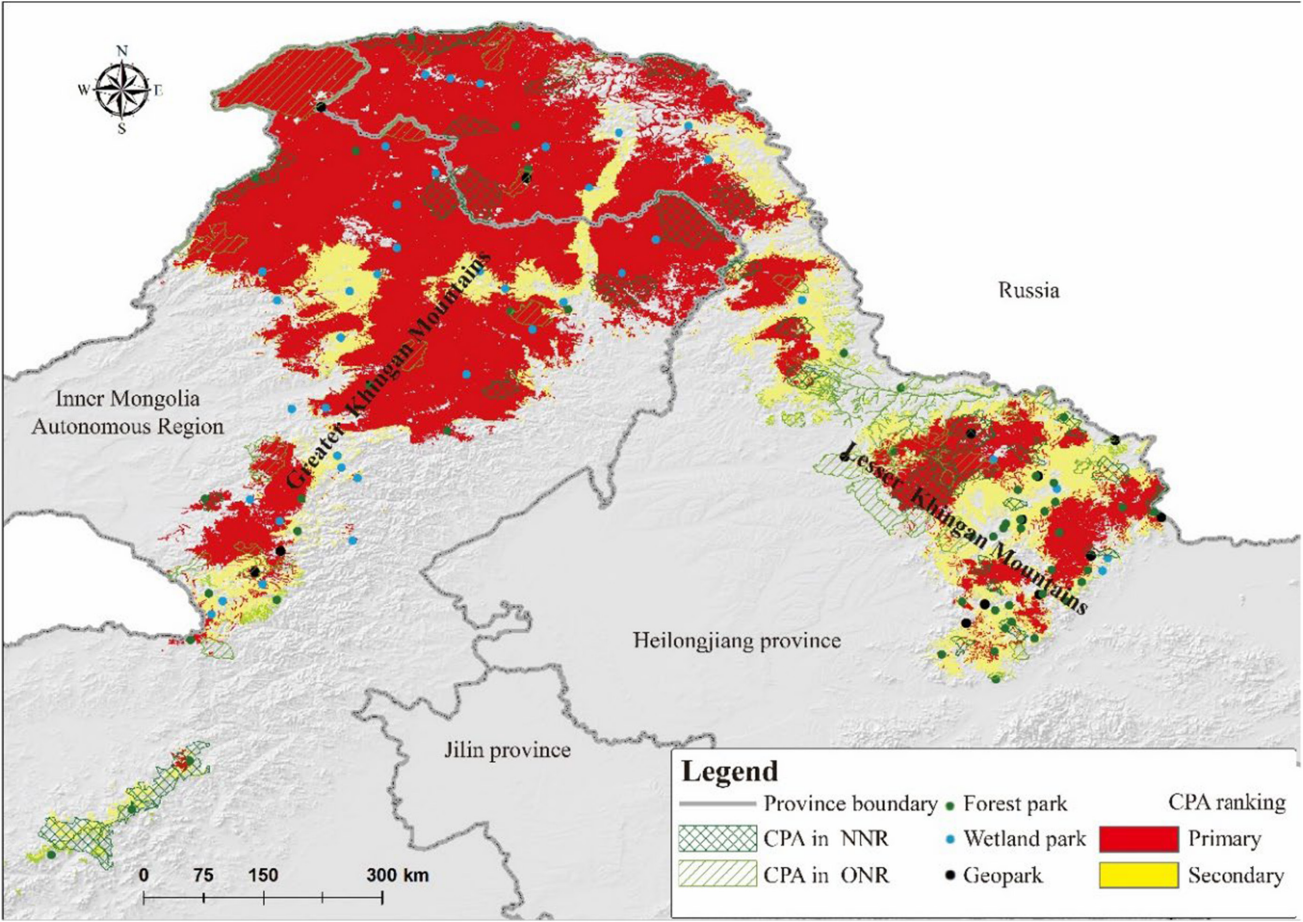
Conservation priority area ranking of the multiple species based on conservation cost. NNR is national nature reserves, ONR is other nature reserves.

The analysis resulted in a CPA covering 220,801 km^2^ of the total study area, primarily in the northeastern part of the GKM, with additional fragments located in the central and eastern parts of the LKM (Figure 3). Approximately, 16.94% of the CPA is currently protected by 35 national nature reserves and 31 provincial and municipal nature reserves. The area of the primary CPA was 168,586 km^2^, and only 31,536 km^2^ (18.71%) was located in nature reserves, of which 17,951 km^2^ was at the national reserves and 13,585 km^2^ was at the provincial and municipal reserves. The area of secondary CPA was 52,215 km^2^, with only 5745 km^2^ (11%) covered by nature reserves, of which the area of national nature reserves was 3776 km^2^, and the area of provincial and municipal nature reserves was 1968 km^2^.

## 4 DISCUSSION

Understanding the determining environmental factors that limit the distribution of species is a good way to identify the greatest risks to their survival, and it is also a prerequisite for the sound implementation of conservation objectives (Xia et al., 2023; Yang et al., 2021). According to our SDM analysis, the environmental variables related to BIO1 (average annual temperature) and anthropogenic disturbance were the most important factors affecting the distribution of wolverine, black‐billed capercaillie, moose, and black grouse. This suggests that in addition to anthropogenic disturbance, climate change (especially climate warming) may impact the population health of species living in the cold temperate zone (Bellard et al., 2012). Recent studies have shown that the geographic distribution of moose has shrunk rapidly over the past 100 years due to a warming climate changing the nutrient composition in the vegetation they consume, driving a trend of rapid northwest retreat (Chen et al., 2022). Anthropogenic pressure was the most important environmental factor affecting the distribution of the more dispersed sable, Siberian musk deer and lynx populations, which followed the contours of low‐intensity anthropogenic disturbance. As a semi‐aquatic mammal, the Eurasian otter was most affected by river and other water‐related environmental variables. Still, anthropogenic pressure and human density were the primary factors affecting its distribution. The results showed that Eurasian otters prefer rivers with less intense human disturbance such as the Gen River, Nuomin River, Jiliu River, and Pangu River in the northern GKM and the Xunbiela River, Gongbiela River, Kuerbin River, and Jiayin River in the LKM. Thus, maintaining the ecological integrity of rivers by reducing anthropogenic disturbance is key for effective Eurasian otter conservation. Compared with other endangered species, the Eurasian otter not only has stronger fecundity but also displays greater environmental tolerance (its distribution being relatively invariant with respect to altitude and temperature), so there is great potential for population recovery (Roos et al., 2015). In December 2016, the Chinese government issued the “Opinions on the Full Implementation of the River Chief System,” which increased protection for aquatic ecosystems and laid the foundation for whole‐basin protection of Eurasian otters. The continuous advancement of ecological protection programs such as the natural forest protection project, the construction of nature reserves in China, and the gradual improvement of legal protections for wildlife has allowed some endangered wild animal populations to recover (Wang et al., 2016). Therefore, in the context of increasing protection for species in the cold temperate zone, in addition to further reducing the negative impact of human activities, a long‐term monitoring mechanism should be established to build a more adaptive protection network.

Conservation plans structured around endangered species protection have been shown to produce a beneficial umbrella effect while remaining efficient with respect to conservation costs (Drummond et al., 2010). In this study, eight endangered species representative of cold temperate forest ecosystems were used to determine CPAs. Based on this analysis, the northern GKM and fragments of the central LKM were listed as primary CPAs. This makes sense for several reasons. First, the northern GKM features the largest contiguous naturally forested area in China (nearly 10,000 km^2^), and there is almost no human activity within this forest. Though most of the area has not yet been incorporated into the nature reserve system, it is still an ideal habitat for wildlife (Pang, 2018). Second, the eight species in this study have a wide historical range but are currently only found in the northern GKM and central LKM. For example, the black‐billed capercaillie occurred across the GKM, LKM, Sanjiang Plain, and Wanda Mountains (Yang, Chen, et al., 2018; Yang, Zhang, et al., 2018;Zhang, Xia, et al., 2020; Zhang, Yang, et al., 2020). The black grouse once reached as far as the Sanjiang Plain and Changbai Mountains (Zhang, Xia, et al., 2020; Zhang, Yang, et al., 2020). The distribution of wolverines once extended to southeastern Heilongjiang Province and eastern Jilin Province (Tian et al., 2003). Moose once occupied the Sanjiang Plain (Xu, 1995). The northern GKM and the central LKM now serve as refugia for these retreating populations. Third, the cost to protect these areas is low. The northern GKMs are sparsely populated, and most of the area is state‐owned forest. As the process of urbanization continues, human disturbance to the habitat of wildlife in this region will likely be reduced in the future (Yang, Chen, et al., 2018; Yang, Zhang, et al., 2018).

## 5 CONCLUSIONS

Based on multiple sources of data to model species occurrence points and multiple environmental variables, we used the SDM (BIOMOD2) to predict the suitable habitat of eight representative species in the Greater and Lesser Khingan Mountains. Their suitable habitat is concentrated in the northern part of the GKM and scattered throughout the central part of the LKM. Zonation was used to identify multispecies CPAs based on conservation costs, and the results showed that the CPAs clustered similar to the PDAs, in the north GKM and the central LKM. However, only 16.94% of the CPAs are covered by national, provincial, or municipal nature reserves. The Greater and Lesser Khingan Mountains may be the last refuge for animals in northeast China. In view of the location's cluster of multiple at‐risk species, low costs of protection, relatively limited human disturbance, and primary forest cover in the northern GKM, we suggest that the Chinese government accelerate the integration of existing protected areas in the northern GKM and establish a larger Greater Khingan Mountains National Park.

## AUTHOR CONTRIBUTIONS

**Chao Zhang:** Conceptualization (lead); data curation (lead); formal analysis (lead); investigation (lead); methodology (lead); resources (equal); software (equal); supervision (equal); validation (equal); visualization (equal); writing – original draft (equal); writing – review and editing (equal). **Zhongwei Lu:** Conceptualization (supporting); data curation (supporting); investigation (supporting); software (equal); writing – original draft (supporting); writing – review and editing (supporting). **Hongfei Zhuang:** Conceptualization (supporting); data curation (supporting); formal analysis (supporting); investigation (equal); methodology (equal); resources (equal); software (equal); visualization (equal); writing – original draft (equal); writing – review and editing (equal). **Jiajie Zhou:** Data curation (equal); investigation (equal); methodology (equal); validation (equal); visualization (equal); writing – review and editing (equal). **Yuan Zhang:** Conceptualization (equal); data curation (equal); investigation (equal); resources (equal); visualization (equal); writing – original draft (equal); writing – review and editing (equal). **Xinyu Lv:** Conceptualization (equal); data curation (equal); investigation (equal); resources (equal); software (equal); writing – original draft (equal). **Minhao Chen:** Data curation (equal); investigation (equal); methodology (equal); resources (equal); software (equal); writing – original draft (equal). **Ali Krzton:** Conceptualization (supporting); writing – original draft (supporting); writing – review and editing (supporting). **Wancai Xia:** Conceptualization (lead); data curation (lead); formal analysis (lead); funding acquisition (lead); investigation (equal); methodology (equal); project administration (lead); resources (lead); software (lead); visualization (lead); writing – original draft (lead); writing – review and editing (lead).

## FUNDING INFORMATION

This study was supported by grants from the National Natural Science Foundation of China (32200401) and Fundamental Research Funds of China West Normal University (21E039).

## CONFLICT OF INTEREST STATEMENT

No conflict of interest exists in the submission of this manuscript, and the manuscript is approved by all authors for publication.

## Supporting information


Data S1.

## Data Availability

Not applicable.
