# Supplementary material for: Identification of potential suitable areas and conservation priority areas for representative wild animals in the Greater and Lesser Khingan Mountains
Source: Ecol Evol. 2024 Jun 18;14(6):e11600. doi: 10.1002/ece3.11600 (PMC11187939; doi:10.1002/ece3.11600)
Supplement: Supplementary file 1 — Data S1. [file ECE3-14-e11600-s001.docx]

**A. Brief description of species ecological habits and selection of environmental variables**

**Black-billed capercaillie** is an avian representative of coniferous forests in the cold temperate zone and was once an important game bird in China. It survives chiefly in the cold-warm coniferous forests and mixed forests in the Greater Khingan Mountains, as the black-billed capercaillie population in most of the Lesser Khingan Mountains is in danger of extinction [1, 2]. Referring to the results of previous ecological studies on black-billed capercaillie, this study selected four climate variables: BIO1 (average annual temperature), BIO12 (annual precipitation), average precipitation in June and average temperature in June; three landform variables: altitude, slope and aspect; one water-related environmental variable: distance from river; four vegetation-related variables: shrub distribution, birch distribution, larch distribution and forest land density; and five anthropogenic disturbance-related environmental variable: distance from the road, residential density, population density, lighting index, anthropogenic pressure. A total of 17 environmental variables were used to model the distribution of black-billed capercaillie. Average precipitation and average temperature in June were highly correlated with the reproductive success of black-billed capercaillie [3, 4], while the birds depend on shrub, birch and larch for both food and habitat [5-7].

**Black grouse** is a typical bird in coniferous forests in cold temperate zones. Due to overhunting and habitat destruction, the population of black grouse in China has declined considerably, becoming endangered in the eastern part of Northeast China [8, 9]. According to the physiological and ecological requirements of black grouse, four climate variables were selected in this study: BIO1 (average annual temperature), BIO12 (annual precipitation), average precipitation and average temperature in June; two landform variables: altitude and slope; one water-related environmental variable: distance from river; five vegetation-related variables: shrub distribution, birch distribution, poplar distribution, larch distribution and forest land density; and five anthropogenic disturbance variables: lighting index, anthropogenic pressure, distance from road, human density and residential density. A total of 17 environmental variables were used to model the distribution of black grouse. Similar to the black-billed capercaillie, the June climate heavily impacts the reproductive success of the black grouse [10, 11], while shrub, birch, poplar, and larch are important habitats for black grouse with abundant food sources [12, 13].

**Wolverine** is a typical Arctic circumpolar mammal, highly sensitive to anthropogenic disturbance. Its distribution in China is the southernmost margin of its geographical distribution, currently coniferous forests and mixed forests in the northern part of the Greater Khingan Mountains. They primarily utilize birch and larch habitats [14]. With reference to previous studies on wolverine habitat selection [14-16], 14 environmental variables were used to model their distribution, including four climate variables: BIO1 (average annual temperature), BIO12 (annual precipitation), BIO17 (precipitation of driest quarter) and BIO19 (precipitation of coldest quarter); one landform variable: altitude; one water-related variable: distance from river; three vegetation-related variables: birch distribution, larch distribution and forest land density; and five anthropogenic disturbance variables: lighting index, anthropogenic pressure, distance from road, human density and residential density.

**Moose** mainly inhabit the Greater Khingan Mountains and Lesser Khingan Mountains in marsh scrub and birch landscapes. Referring to previous studies on moose habitat selection [17-19], this study selected 13 environmental variables to simulate its distribution, including two climate variables: BIO1 (average annual temperature) and BIO12 (annual precipitation); two landform variables: altitude and aspect; one water-related variable: distance from river; three vegetation-related variables: birch distribution, shrub distribution and forest land density; and five anthropogenic disturbance variables: lighting index, anthropogenic pressure, distance from road, human density and residential density.

In China, **sable** mainly lives in mixed broadleaf and deciduous broadleaf forests in northeast China and northern Xinjiang. As it is not very sensitive to vegetation type, no environmental variables related to vegetation type were selected for this study [20, 21]. Instead, referring to previous studies [20, 21], the 14 environmental variables for modeling the distribution of sable included four climate variables: BIO1 (average annual temperature), BIO12 (annual precipitation), BIO17 (precipitation of driest quarter) and BIO19 (precipitation of coldest quarter); three landform variables: altitude, aspect and slope; one water-related variable: distance from river; one vegetation-related variable: forest land density; and five anthropogenic disturbance variables: lighting index, anthropogenic pressure, distance from road, human density and residential density.

Habitat fragmentation has become a barrier to the population recovery of **Siberian musk deer** [22]. In this study, a total of 13 environmental variables were selected for species distribution modeling based on the IUCN Species Assessment report and published studies on Siberian musk deer habitat selection [23, 24], including two climate variables: BIO1 (annual average temperature) and BIO12 (annual precipitation); two landform variables: altitude and slope; four vegetation-related variables: birch distribution, larch distribution, shrub distribution and forest land density; and five anthropogenic disturbance variables: lighting index, anthropogenic pressure, distance from road, human density and residential density.

**Lynx** is a carnivorous cat widely distributed in northeast China. Its presence is crucial to maintaining ecological balance. Similar to **sable**, vegetation type is not an important determinant of lynx distribution. Referring to previous studies on lynx habitat selection [25, 26], 14 environmental variables were selected for distribution modeling including five climate variables: BIO1 (average annual temperature), BIO12 (annual precipitation), BIO5 (max temperature of warmest month), BIO6 (min temperature of coldest month), BIO16 (precipitation of wettest quarter); two topographic variables: altitude and surface relief; one water-related variable: distance from river; one vegetation-related variable: forest land density; and five anthropogenic disturbance variables: lighting index, anthropogenic pressure, distance from road, human density and residential density.

**Eurasian otter** is highly sensitive to water quality. This carnivorous mammal is an indicator and flagship species of aquatic ecosystems [27, 28]. Referring to previous studies and combined with the ecological profile of otters, 13 environmental factors were selected to model the distribution of Eurasian otter [27-30], including five water-related variables: BIO1 (annual precipitation), BIO16 (precipitation of wettest quarter), BIO17 (precipitation of driest quarter), distance from river, and humidity index; two topographic variables: altitude and surface relief; one vegetation-related variable: forest land density; and five anthropogenic disturbance variables: lighting index, anthropogenic pressure, distance from road, human density and residential density.

**B.** **Distribution points of eight species in the Greater and Lesser Hingan Mountains**

**
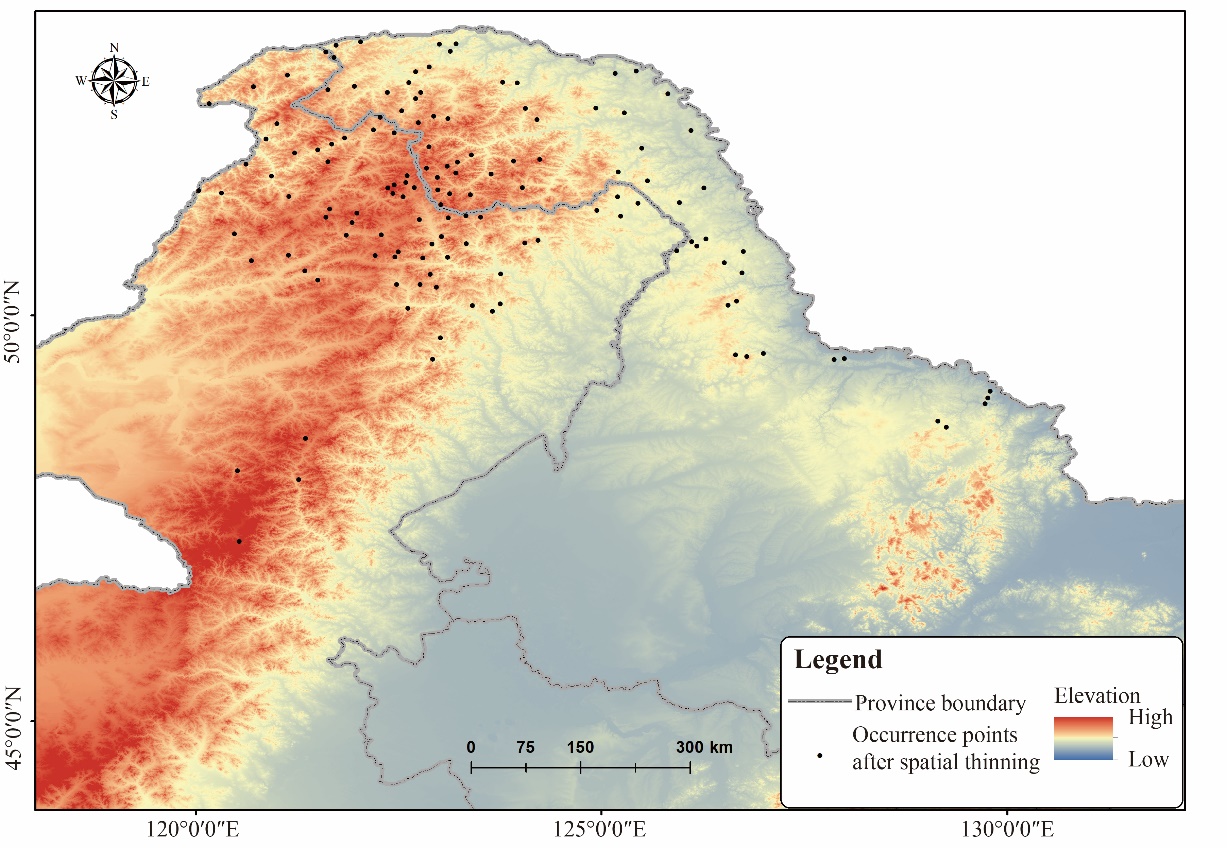
**

Black-billed capercaillie


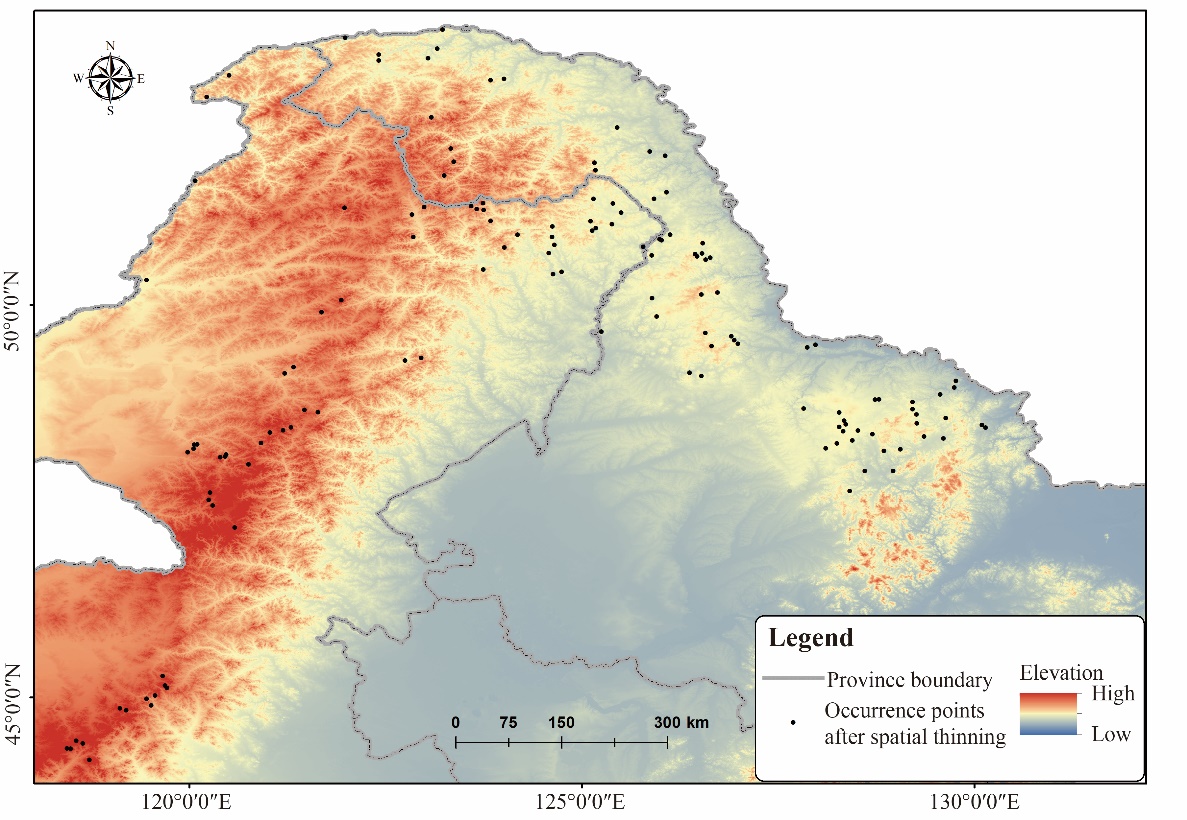


Black grouse


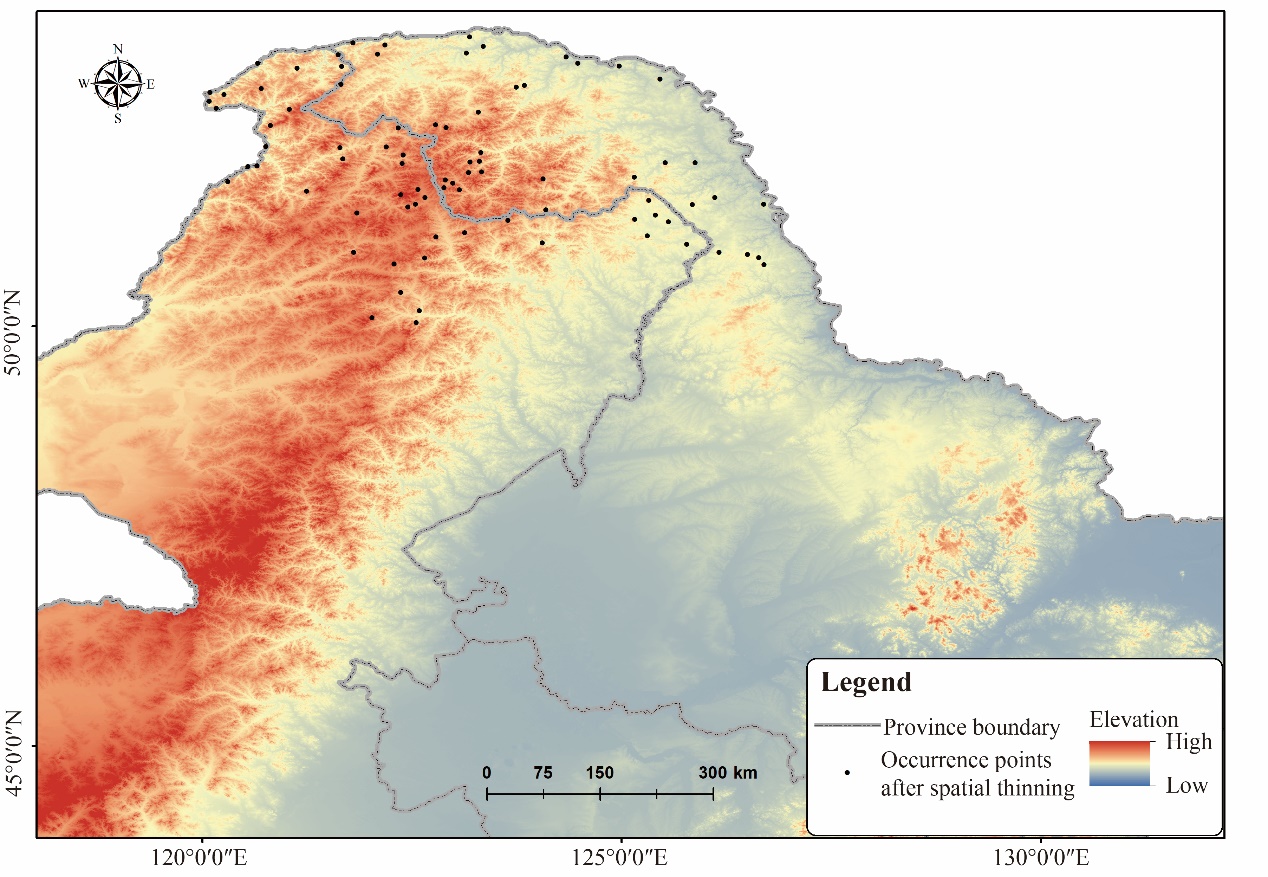


Wolverine


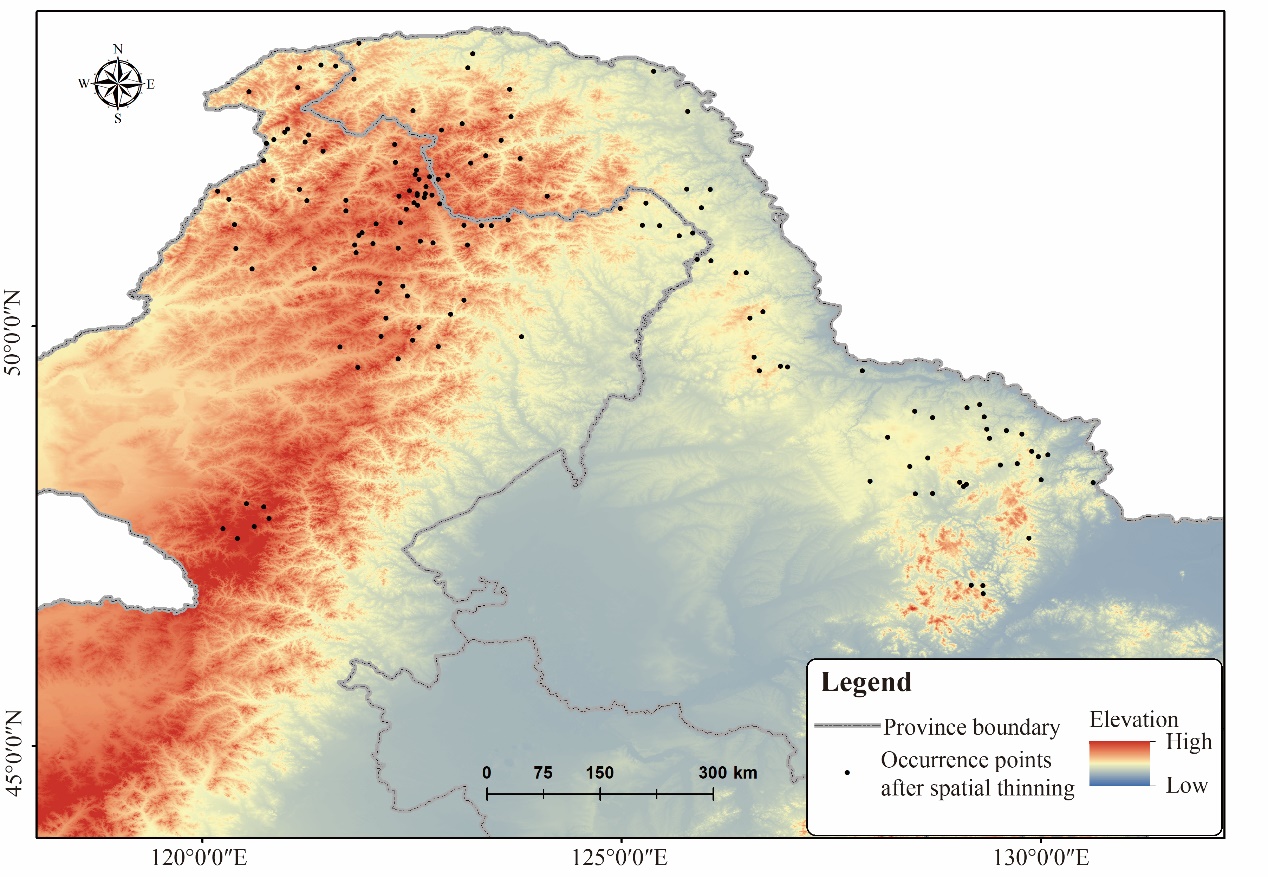


Moose


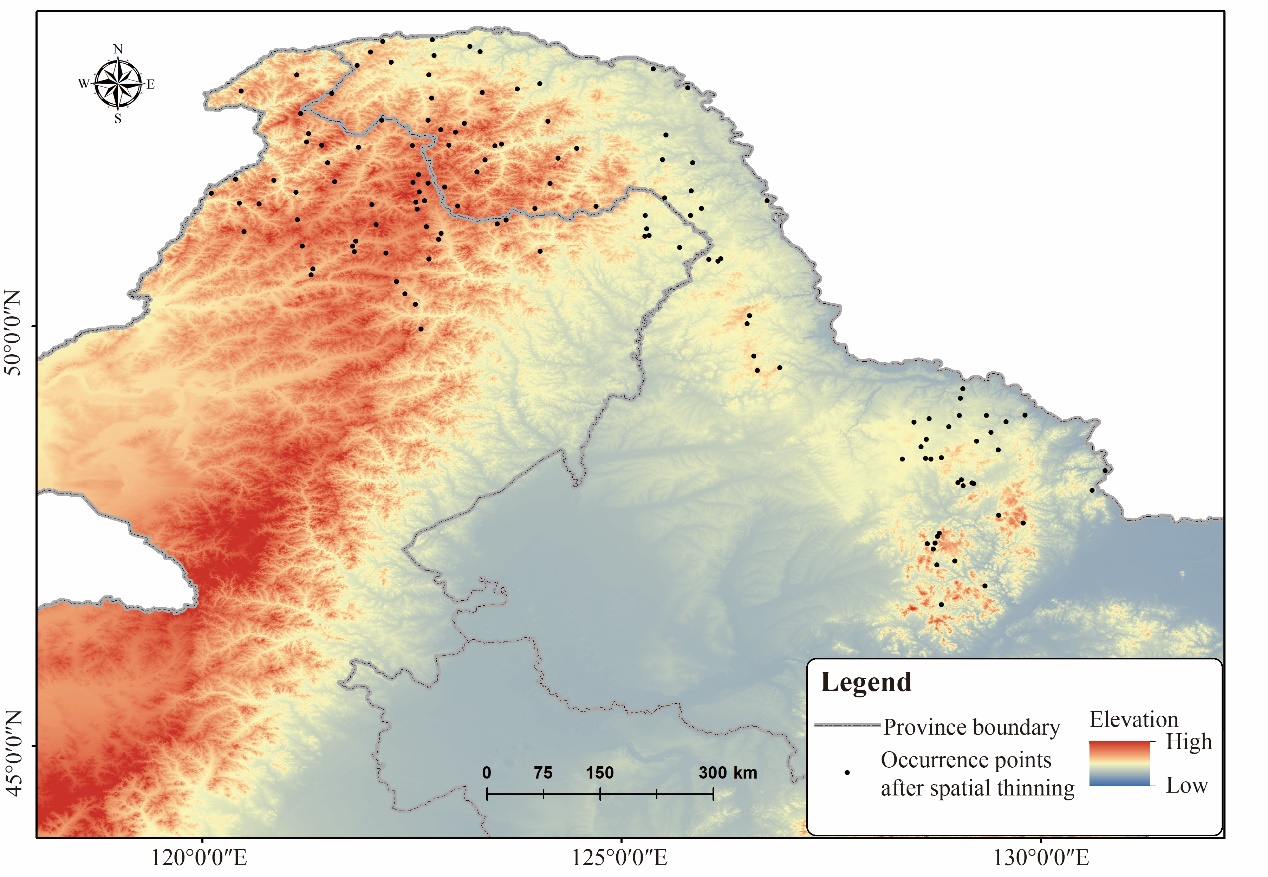


Sable


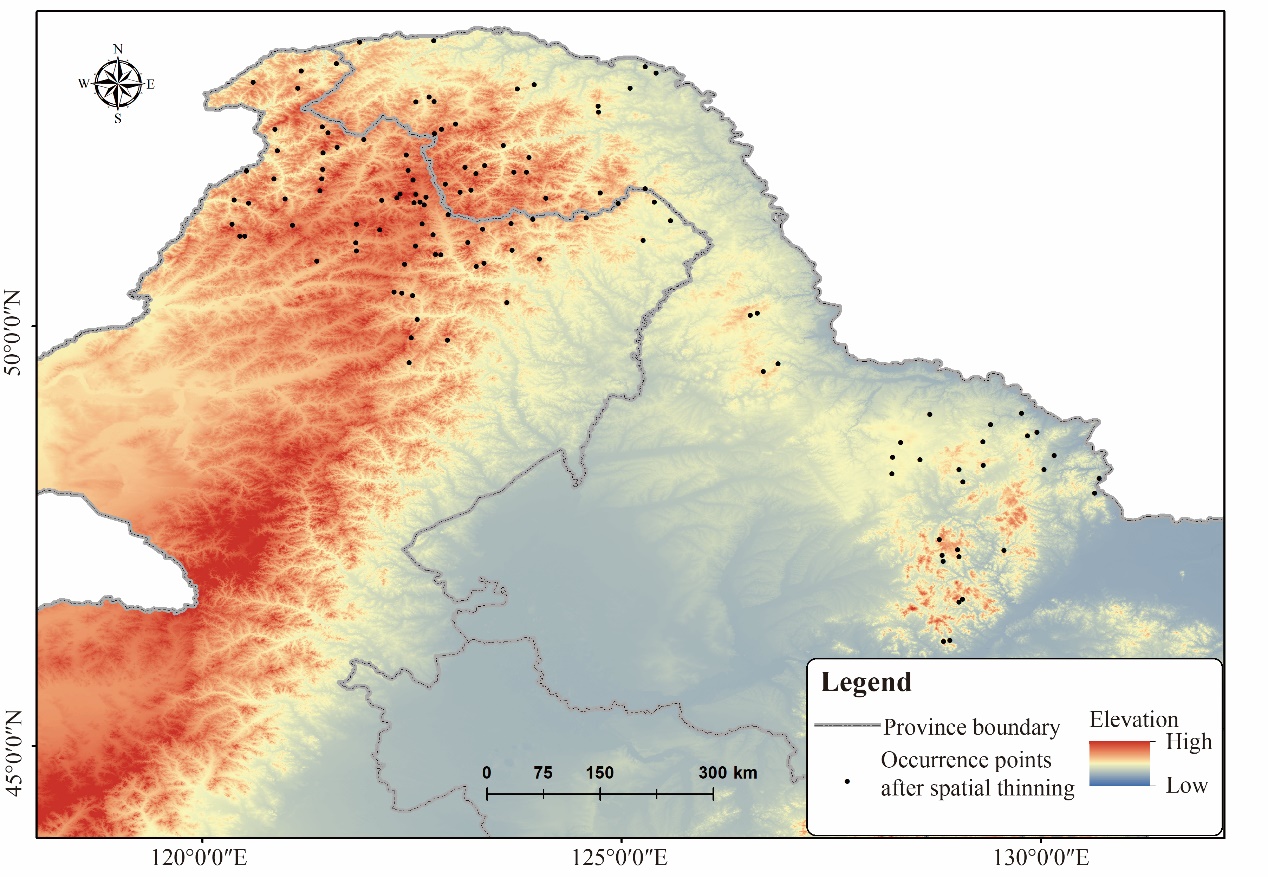


Siberian musk deer


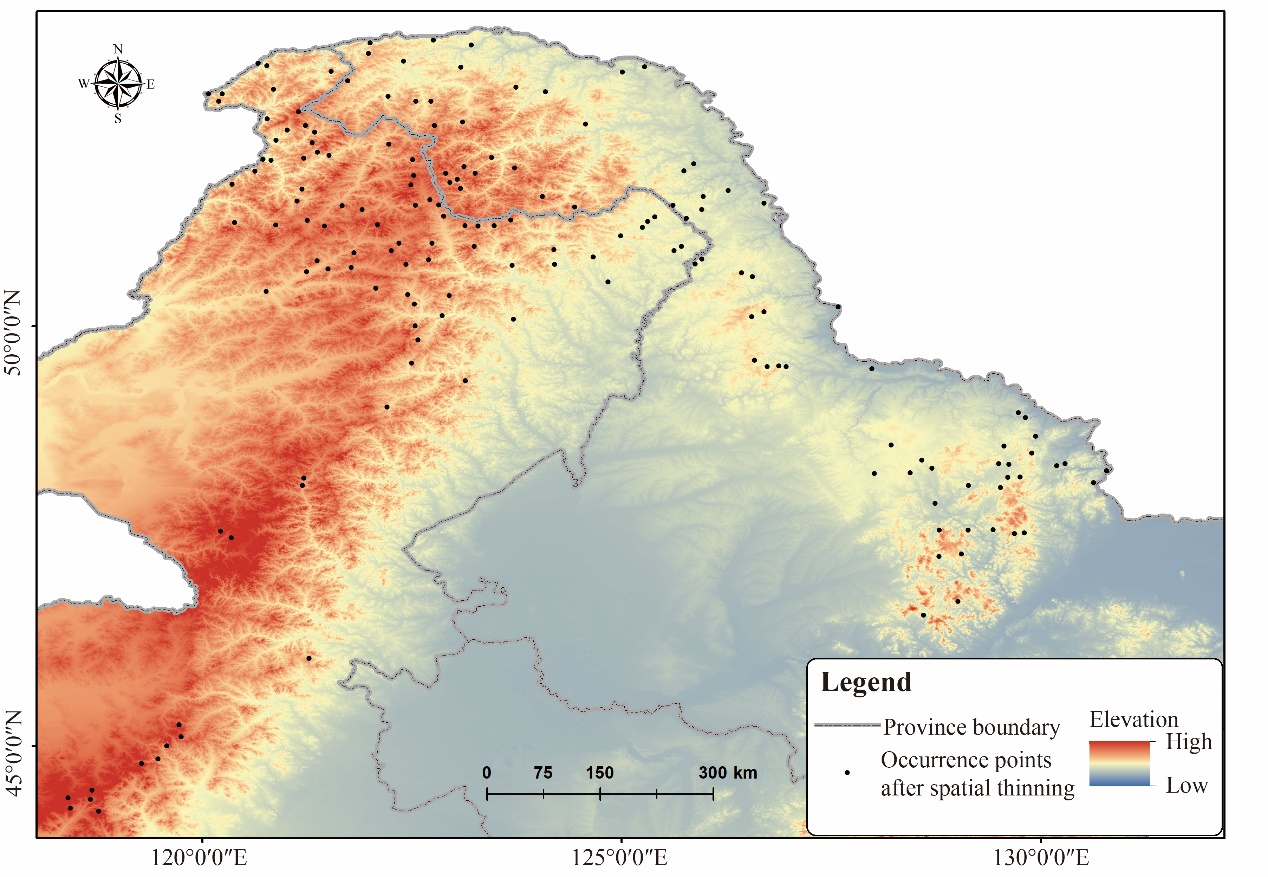


Lynx


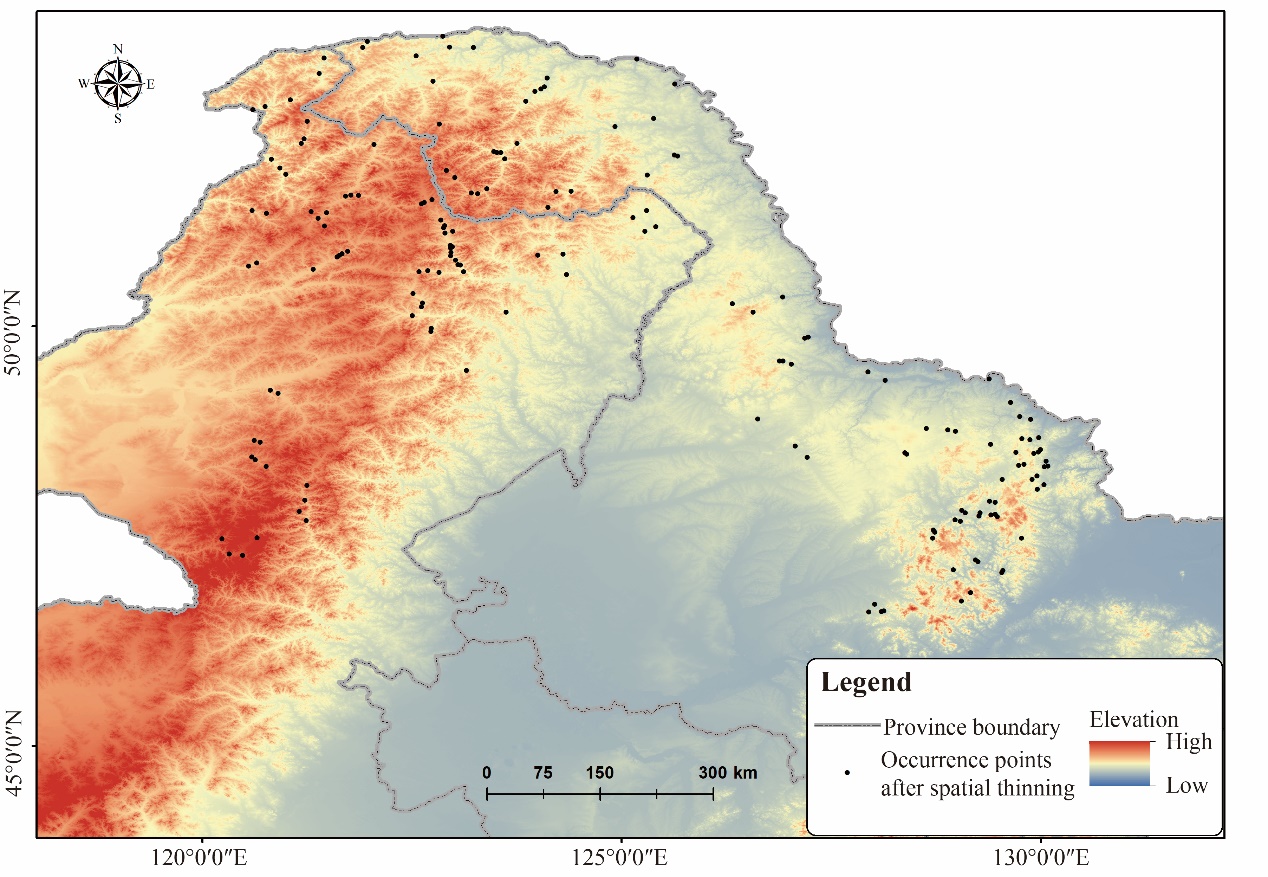


Eurasian otter

**C Variance inflation factor value and importance of environmental variables**

Table C-1 Variance inflation factor value and importance of environmental variables of the black-billed capercaillie

| **Variables** | **First** **VIF evaluation** | **Second VIF evaluation** | **Importance** |
| --- | --- | --- | --- |
| BIO1 | 16.086 | 6.366 | 0.083 |
| BIO12 | 7.536 | 4.943 | 0.050 |
| Average precipitation in June | 5.366 | 4.633 | 0.007 |
| Average temperature in June | 20.475 | - | - |
| Birch distribution | 1.035 | 1.034 | 0.006 |
| Larch distribution | 2.354 | 2.301 | 0.047 |
| Shrub distribution | 3.532 | 3.284 | 0.127 |
| Forest land density | 4.142 | 4.113 | 0.006 |
| Altitude | 5.296 | 3.488 | 0.007 |
| Aspect | 1.035 | 1.034 | 0.006 |
| Slope | 1.063 | 1.059 | 0.027 |
| Distance from the road | 1.517 | 1.517 | 0.002 |
| Distance from river | 1.463 | 1.415 | 0.001 |
| Residential density | 3.854 | 3.759 | 0.002 |
| Human density | 1.306 | 1.301 | 0.041 |
| Lighting index | 1.507 | 1.507 | 0.010 |
| Anthropogenic pressure | 3.324 | 3.207 | 0.083 |

Table C-2 Variance inflation factor value and importance of environmental variables of the black grouse

| **Variables** | **First VIF evaluation** | **Second VIF evaluation** | **Importance** |
| --- | --- | --- | --- |
| BIO1 | 16.607 | 7.391 | 0.196 |
| BIO12 | 7.537 | 4.936 | 0.022 |
| Average precipitation in June | 5.845 | 5.021 | 0.018 |
| Average temperature in June | 20.582 | — | — |
| Birch distribution | 4.277 | 4.027 | 0.006 |
| Larch distribution | 2.402 | 2.341 | 0.047 |
| Poplar distribution | 1.827 | 1.815 | 0.032 |
| Shrub distribution | 3.581 | 3.318 | 0.016 |
| Forest land density | 4.154 | 4.128 | 0.013 |
| Altitude | 5.529 | 3.626 | 0.007 |
| Slope | 1.051 | 1.047 | 0.011 |
| Distance from the road | 1.518 | 1.518 | 0.01 |
| Distance from river | 1.498 | 1.444 | 0.004 |
| Residential density | 1.827 | 3.797 | 0.009 |
| Human density | 1.305 | 1.301 | 0.07 |
| Lighting index | 1.51 | 1.51 | 0.016 |
| Anthropogenic pressure | 3.326 | 3.216 | 0.109 |

Table C-3 Variance inflation factor value and importance of environmental variables of the wolverine

| **Variables** | **VIF** | **Importance** |
| --- | --- | --- |
| BIO1 | 4.201 | 0.343 |
| BIO12 | 6.687 | 0.031 |
| BIO16 | 5.915 | 0.04 |
| BIO17 | 4.309 | 0.013 |
| Birch distribution | 3.039 | 0.012 |
| Larch distribution | 2.213 | 0.011 |
| Forest land density | 3.943 | 0.002 |
| Altitude | 1.964 | 0.055 |
| Distance from the road | 1.503 | 0.017 |
| Distance from river | 1.41 | 0.001 |
| Residential density | 3.678 | 0.064 |
| Human density | 1.299 | 0.032 |
| Lighting index | 1.523 | 0.009 |
| Anthropogenic pressure | 3.088 | 0.032 |

Table C-4 Variance inflation factor value and importance of environmental variables of the moose

| **Variables** | **VIF** | **Importance** |
| --- | --- | --- |
| BIO1 | 5.865 | 0.166 |
| BIO12 | 2.671 | 0.075 |
| Birch distribution | 2.873 | 0.033 |
| Shrub distribution | 3.165 | 0.054 |
| Forest land density | 3.972 | 0.03 |
| Altitude | 3.421 | 0.01 |
| Slope | 1.043 | 0.002 |
| Distance from the road | 1.508 | 0.002 |
| Distance from river | 1.396 | 0.005 |
| Residential density | 3.455 | 0.014 |
| Human density | 1.293 | 0.053 |
| Lighting index | 1.5 | 0.015 |
| Anthropogenic pressure | 3.154 | 0.124 |

Table C-5 Variance inflation factor (VIF) value and importance of environmental variables of the sable

| **Variables** | **VIF** | **Importance** |
| --- | --- | --- |
| BIO1 | 2.753 | 0.071 |
| BIO12 | 6.532 | 0.076 |
| BIO16 | 5.671 | 0.121 |
| BIO19 | 4.167 | 0.032 |
| Forest land density | 3.078 | 0.041 |
| Altitude | 1.948 | 0.013 |
| Aspect | 1.03 | 0.002 |
| Slope | 1.06 | 0.025 |
| Distance from the road | 1.502 | 0.005 |
| Distance from river | 1.37 | 0.007 |
| Residential density | 3.659 | 0.015 |
| Human density | 1.296 | 0.092 |
| Lighting index | 1.516 | 0.01 |
| Anthropogenic pressure | 3.132 | 0.321 |

Table C-6 Variance inflation factor value and importance of environmental variables of the Siberian musk deer

| **Variables** | **VIF** | **Importance** |
| --- | --- | --- |
| BIO1 | 6.261 | 0.052 |
| BIO12 | 2.644 | 0.237 |
| Birch distribution | 2.854 | 0.012 |
| Larch distribution | 2.118 | 0.022 |
| Shrub distribution | 3.177 | 0.005 |
| Forest land density | 4.095 | 0.046 |
| Altitude | 3.328 | 0.015 |
| Slope | 1.044 | 0.021 |
| Distance from the road | 1.508 | 0.002 |
| Residential density | 3.442 | 0.053 |
| Human density | 1.295 | 0.006 |
| Lighting index | 1.504 | 0.001 |
| Anthropogenic pressure | 3.145 | 0.237 |

Table C-7 Variance inflation factor value and importance of environmental variables of the lynx

| **Variables** | **First VIF evaluation** | **Second VIF evaluation** | **Importance** |
| --- | --- | --- | --- |
| BIO1 | 29.159 | — | — |
| BIO12 | 6.48 | 6.324 | 0.125 |
| BIO16 | 4.346 | 4.079 | 0.014 |
| BIO5 | 7.984 | 5.083 | 0.026 |
| BIO6 | 14.879 | 3.228 | 0.025 |
| Forest land density | 3.525 | 3.284 | 0.051 |
| Altitude | 8.388 | 8.106 | 0.025 |
| Surface relief | 6.465 | 6.437 | 0.005 |
| Distance from the road | 1.51 | 1.507 | 0.005 |
| Distance from river | 1.411 | 1.408 | 0.004 |
| Residential density | 3.564 | 3.538 | 0.017 |
| Human density | 1.306 | 1.304 | 0.134 |
| Lighting index | 1.512 | 1.507 | 0.004 |
| Anthropogenic pressure | 3.151 | 3.091 | 0.228 |

Table C-8 Variance inflation factor value and importance of environmental variables of the otter

| **Variables** | **First VIF evaluation** | **Second VIF evaluation** | **Importance** |
| --- | --- | --- | --- |
| BIO12 | 16.097 | — | — |
| BIO16 | 4.444 | 2.909 | 0.098 |
| BIO17 | 6.447 | 3.208 | 0.017 |
| Forest land density | 2.977 | 2.519 | 0.038 |
| Humidity index | 3.373 | 3.062 | 0.072 |
| Altitude | 7.398 | 7.15 | 0.03 |
| Surface relief | 6.477 | 6.404 | 0.026 |
| Distance from the road | 1.493 | 1.493 | 0.002 |
| Distance from river | 1.364 | 1.358 | 0.559 |
| Residential density | 3.032 | 2.691 | 0.008 |
| Human density | 1.285 | 1.282 | 0.049 |
| Lighting index | 1.504 | 1.498 | 0.001 |
| Anthropogenic pressure | 3.046 | 3.032 | 0.06 |

BIO1, average annual temperature; BIO5, max temperature of warmest month; BIO6, min temperature of coldest month; BIO12, annual precipitation; BIO16, precipitation of wettest quarter; BIO17, precipitation of driest quarter; BIO19, precipitation of coldest quarter.

**D The interviews, questionnaire surveys and field investigations**

The interviewees were local community residents and wildlife lovers, including 30 people from Inner Mongolia, 115 people from Heilongjiang. The specific method of interview survey is to use the remote sensing image map of the survey site, and the interviewees mark the occurrence points of species on the map, and then complete the digitization on the Google remote sensing image to obtain the longitude and latitude coordinates of species distribution. The questionnaire was issued by Inner Mongolia Forestry Industry Group, Greater Xinganling Forestry Group, Heihe Forestry and Grassland Bureau, Yichun Forest Industry Group. A total of 57 questionnaires were issued and 40 valid questionnaires were collected, covering almost all typical forest ecosystems in the study area spatially. The species survey questionnaire is in Appendix C. The distribution sites of all species were verified according to their suitable habitats in the Google remote sensing image (based on 91 map software v18.8.5), and cross-verified using local Chronicles, zoology, and published academic papers, so as to exclude doubtful and unreliable species distribution data. In the field investigation, the species feces and solid sites were recorded by line transect survey, combined with the results of infrared camera survey in this area.

**References**

1. Gao, ZX., Liu, GY., Zhao, YJ. (1982). Ecology of *Tetrao parvirostris*. Wildlife *3*, 29-32(In Chinese).

2. Yin, XY., Ge, DN., Guan, XM., Sun, HY., Li ZH. (2009). Population and habitat status of black-billed capercaillie in the Northeast of China. Territory & Natural Resources Study *2*, 90-91(In Chinese).

3. Graf, R., Bollmann, K., Suter, W., and Bugmann, H. (2005). The Importance of Spatial Scale in Habitat Models: Capercaillie in the Swiss Alps. Landscape Ecology *20*, 703-717.

4. Wegge, P., and Rolstad, J. (2011). Clearcutting forestry and Eurasian boreal forest grouse: Long-term monitoring of sympatric capercaillie Tetrao urogallus and black grouse T. tetrix reveals unexpected effects on their population performances. Forest Ecology and Management *261*, 1520-1529.

5. Lu, TC. (1991). The rare and endangered gamebirds in China. Fujian Science and Technology Press(In Chinese).

6. Gao, ZX., Jin, K. (1995). Wildlife Animals in Small Xing 'an Mountain. Hilongjiang Science and Technology Press (In Chinese).

7. IUCN (2021). The IUCN Red List of Threatened Species 2021[OL]. Volume 2021-03. p. <https://www.iucnredlist.org/>.

8. Yin, XY., Tian, JL. (2009). Population status of black grouse and its protection and management countermeasures in Chnia. China forest vice specialty *1*, 86-87 (In Chinese).

9. Liang, BK., Wwang, ZC., Sun, HY., Tian, JL. (2012). Population Status and Nest-Site Habitat Evaluation of Black Grouse (*Lyrurus tetrix*) in Its Eastern Distribution Areas in China. Journal of Northeast Forestry University *40*, 123-127 (In Chinese).

10. Summers, R., Dugan, D., and Proctor, R. (2010). Numbers and breeding success of Capercaillies Tetrao urogallus and Black Grouse T. tetrix at Abernethy Forest, Scotland. Bird Study *57*, 437-446.

11. Summers, R., Green, R., Proctor, R., Dugan, D., Lambie, D., Moncrieff, R., Moss, R., and Baines, D. (2004). An experimental study of the effects of predation on the breeding productivity of capercaillie and black grouse. Journal of Applied Ecology *41*, 513-525.

12. Brittas, R., and Willebrand, T. (1991). Nesting Habitats and Egg Predation in Swedish Black Grouse. Ornis Scandinavica *22*, 261-263.

13. Patthey, P., Signorell, N., Rotelli, L., and Arlettaz, R. (2011). Vegetation structural and compositional heterogeneity as a key feature in Alpine black grouse microhabitat selection: conservation management implications. European Journal of Wildlife Research *58*, 59-70.

14. Zhu,SB., Zhang, MH., Li, HJ., Liu, YJ. (2009). Winter habitat selection of wolverine in Greater Khingan Mountains. Wildlife *30*, 115-117(In Chinese).

15. Tian,JL., Piao, RZ., Liu, JS., Liu, QW. (1999). habitat selection of wolverine in Greater Khingan Mountains. China forest vice specialty *4*, 54-55(In Chinese).

16. May, R., Landa, A., van Dijk, J., Linnell, J., and Andersen, R. (2006). Impact of infrastructure on habitat selection of wolverines (*Gulo gulo*). Wildlife Biology *12*, 285-295.

17. Dussault, C., Courtois, R., and Ouellet1, J. (2006). A habitat suitability index model to assess moose habitat selection at multiple spatial scales. Canadian Journal of Forest Research *36*, 1097-1107.

18. Yu, YZ., Li, Q., Zhang, MH., Li, BG. (2019). Multi-scale Model in Animal Habitat Selection: A Case Study of Moose. Journal of Northeast Forestry University *47*, 58-61(In Chinese).

19. Yu, YZ., Li, Q., Zhang, MH., Du, HR., Li, Q., Zhang, LB., et al. (2019). Optimized MAXENT Model in Simulating Distribution of Suitable Habitat of Moose. Journal of Northeast Forestry University *47*, 81-84(In Chinese).

20. Li, HJ., Zhu, SB., Zhang, SF., Zhao, XD., Chen, P. (2016). Winter Habitat Evaluation of Sableinthe northern region of the Greater Hing`an Mountains. Territory &Natural Resources Study *5*, 71-75(In Chinese).

21. Sheng, Q., Dong, LB., Liu, ZG. (2020). Suitability Assessment of Wild Animal Habitatwith GIS--Take Martes zibellinain Pangu Forest Farm of Daxing’an Mountains as An Example. Journal of Northeast Forestry University *48*, 157-162(In Chinese).

22. Yang, Q., Meng, X., Xia, L., and Feng, Z. (2003). Conservation status and causes of decline of musk deer (*Moschus spp*.) in China. Biological Conservation *109*, 333-342.

23. Zhang, DD., Zhu, HQ., Jiang, CY., Li, C., Zhang, XD., Liu, CF., et al. (2014). Ｒesearch Situation of Impact Factors on Habitat Selection of Siberian *Moschus moschiferus*. Journal of Economic Animal *18*, 44-46(In Chinese).

24. Slaght, J., Milakovsky, B., Maksimova, D., Seryodkin, I., Zaitsev, V., and Miquelle, D. (2017). Anthropogenic influences on the distribution of a Vulnerable coniferous forest specialist: habitat selection by the Siberian musk deer *Moschus moschiferus*. Oryx *53*, 174-180.

25. Zimmermann, F., and Breitenmoser, U. (2007). Potential distribution and population size of the Eurasian lynx (*Lynx lynx*) in the Jura Mountains and possible corridors to adjacent ranges. Wildlife Biology *13*, 406-416.

26. Alfaya, P., Casanovas, J., Lobón-Rovira, J., Matallanas, B., Cruz, A., Arana, P., and Alonso, G. (2019). Using MaxEnt algorithm to assess habitat suitability of a potential Iberian lynx population in central Iberian Peninsula. Community Ecology *20*, 266-276.

27. Cianfrani, C., Lay, G., Maiorano, L., Satizábal, H., Loy, A., and Guisan, A. (2011). Adapting global conservation strategies to climate change at the European scale: The otter as a flagship species. Biological Conservation *144*, 2068-2080.

28. Zhang, L., Wang, Q., Yang, L., Li, F., and Fan, P. (2018). The neglected otters in China: Distribution change in the past 400 years and current conservation status. Biological Conservation *228*, 259-267.

29. Robitaille, J., and Laurence, S. (2002). Otter, Lutra lutra, occurrence in Europe and in France in relation to landscape characteristics. Animal Conservation *5*, 337-344.

30. Remonti, L., Balestrieri, A., and Prigioni, C. (2009). Altitudinal gradient of Eurasian otter (*Lutra lutra*) food niche in Mediterranean habitats. Canadian Journal of Zoology *87*, 285-291.
